# Early Acute Kidney Injury in Stroke Patients Submitted to Endovascular Treatment: A Cohort Study

**DOI:** 10.3390/jcm13226726

**Published:** 2024-11-08

**Authors:** Marta Oliveira, Miguel Sousa, Rita Antunes, Diogo Macedo, Sabina Belchior, Daniela Soares, Francisco de Oliveira Simões, Mariana Rocha, Henrique Costa, Joana Novo, Ludovina Paredes, Pedro Barros, Pedro Pires, Sérgio Castro, Manuel Ribeiro, André Araújo, Vera Afreixo, Tiago Gregorio

**Affiliations:** 1 Department of Internal Medicine, Hospital CUF Porto, Estrada da Circunvalação 14341, 4100-180 Porto, Portugal; martasoliv@gmail.com; 2 Department of Internal Medicine, Unidade Local de Saúde de Gaia e Espinho EPE, Rua Conceição Fernandes, 4434-502 Vila Nova de Gaia, Portugal; miguelangelocmsousa@gmail.com (M.S.); ritaantunes811@gmail.com (R.A.); diogobmacedo16@gmail.com (D.M.); ludoparedes@gmail.com (L.P.); 3 Department of Internal Medicine, Unidade Local de Saúde do Alto Minho, Estrada de Santa Luzia 50, 4900-408 Viana do Castelo, Portugal; sabinadazevedo@gmail.com; 4 Department of Internal Medicine, Unidade Local de Saúde de Entre-Douro-e-Vouga, Rua Dr. Cândido Pinho 5, 4520-211 Santa Maria da Feira, Portugal; danielafilipa575@gmail.com; 5 Department of Internal Medicine, Unidade Local de Saúde de Braga, Rua das Sete Fontes, 4710-243 Braga, Portugal; francisco.simoes.t@gmail.com; 6 Stroke Unit, Unidade Local de Saúde de Gaia e Espinho EPE, Rua Conceição Fernandes, 4434-502 Vila Nova de Gaia, Portugal; mariana.ag.rocha@gmail.com (M.R.); henriq.costa@gmail.com (H.C.); jfscnovo@gmail.com (J.N.); pedrojgbarros@gmail.com (P.B.); 7 Cerebrovascular Interventional Neuroradiology Unit, Unidade Local de Saúde de Gaia e Espinho EPE, Rua Conceição Fernandes, 4434-502 Vila Nova de Gaia, Portugal; pedro.pires@ulsge.min-saude.pt (P.P.); snacastro@gmail.com (S.C.); mqribeiro@gmail.com (M.R.); andrearaujo27@gmail.com (A.A.); 8 Center for Research and Development in Mathematics and Applications, University of Aveiro, Campus Universitário de Santiago, 3810-193 Aveiro, Portugal; vera@ua.pt; 9 CINTESIS, Faculty of Medicine, University of Porto, Rua Dr. Plácido Costa, 4200-450 Porto, Portugal

**Keywords:** stroke, thrombectomy, acute kidney injury, risk factors, morbidity, mortality

## Abstract

**Background/Objectives:** Acute kidney injury (AKI) is a potential complication of cardiovascular disorders and is associated with worse outcome. The aim of this study was to assess the incidence of early AKI after endovascular therapy for acute ischemic stroke, identify predictors for this complication, and test the association between AKI and mortality or death or dependency. **Methods:** This was a single-center cohort study involving consecutive patients with acute ischemic stroke submitted to endovascular therapy between 2015 and 2022. AKI was defined according to the KDIGO criteria and evaluated at 48 h. Other outcomes of interest were vital status and functional dependency at 3 months using the modified Rankin Scale, with death or dependency being defined as a score > 2. An adjustment for potential confounders was performed using logistic regression. **Results:** Overall, 1150 patients were included in the analysis, with a mean age of 74 years and a slight female preponderance (56%). The median NIHSS was 15, the mean onset-to-groin time was 392 min, and 92% of patients were successfully recanalized. The overall incidence rate of AKI was 6%. On univariate analysis, patients with AKI were older (*p* = 0.002), had a longer time to EVT (*p* = 0.042), higher NIHSS (*p* = 0.006), higher blood glucose (*p* = 0.033), and lower baseline glomerular filtration rate (GFR) (*p* < 0.001). After adjustment for confounders, AKI was independently associated with NIHSS (*p* = 0.012), time to treatment (*p* = 0.004), and lower baseline GFR (*p* < 0.001). AKI was also independently associated with higher mortality (OR = 2.302, *p* = 0.003). **Conclusions:** Patients with impaired baseline renal function and more severe stroke are at higher risk of AKI, and AKI begets worse stroke outcome. Better strategies are required to optimize treatment outcome in these patients and avert this vicious cycle.

## 1. Introduction

Stroke is a major public health problem and cause of morbidity and mortality. Every year, more than three million people die of ischemic stroke and more than 60 million disability-adjusted life years are lost [1]. Endovascular therapy (EVT) is an approved and highly effective treatment for acute stroke [2,3,4], and its use is expected to increase in the near future due to increasing stroke incidence, increased access to treatment, and expanding treatment indications [5]. EVT requires the administration of contrast media, a nephrotoxic agent with the potential to cause acute kidney injury (AKI) [6,7]. AKI is a clinical syndrome characterized by an abrupt decrease in kidney function that leads to the accumulation of nitrogenous waste products and the dysregulation of extracellular volume and electrolytes [8]. Other factors besides contrast can also lead to AKI (renal hypoperfusion or direct injury to the glomeruli, tubules, and interstitium); however, regardless of the cause, the development of AKI is associated with adverse outcomes in both stroke [9] and non-stroke patients [10]. In stroke patients, the pathways leading to worse outcome probably involve increased inflammation, blood–brain barrier disruption, and contrast media neurotoxicity [11]. Given the association between AKI and stroke outcome, the present study was undertaken to (i) ascertain the incidence of early (≤48 h) AKI in stroke patients submitted to EVT; (ii) determine the risk factors for AKI in this patient population; and (iii) test the hypothesis that AKI is associated with adverse outcome after thrombectomy for stroke.

## 2. Materials and Methods

### 2.1. Population, Sampling, and Recruitment

In this retrospective cohort study, all patients treated with EVT at a European Stroke Organization-certified high-volume stroke center that provides care to a population of approximately 700,000 inhabitants were consecutively assessed for eligibility. Patients were included if they were adult patients (≥18 years) treated with EVT for acute ischemic stroke and had at least two serum creatinine determinations in the first 48 h after symptom onset. Patients on chronic renal replacement therapy were excluded from the study, as dialysis affects the method used for the assessment of the exposure of interest.

### 2.2. Outcomes and Predictors

The outcomes of interest were acute kidney injury, mortality, and death or dependency at three months. AKI was defined as an increase in serum creatinine of at least 0.3 mg/dL or ≥50% from baseline within 48 h after symptom onset, based on the KDIGO guidelines [12]. Functional status was assessed at three months on outpatient visits using the modified Rankin Scale (mRS) [13], with death or dependency being defined as a score of three or higher. In addition to the outcomes of interest, the following predictors were also retrieved prior to EVT from the local electronic health records: age, sex at birth, stroke severity according to the NIHSS stroke scale [14], site of vessel occlusion (anterior or posterior circulation), thrombolysis, time to EVT (onset-to-groin time), successful recanalization (defined as a modified TICI score ≥ 2b) [15], stenting, stroke etiology according to the TOAST classification [16], history of hypertension, diabetes, dyslipidemia or active smoking, blood glucose, systolic blood pressure, diastolic blood pressure, and baseline glomerular filtration rate according to the CKD-EPI equation [17]. Onset-to-groin time, NIHSS and baseline renal glomerular filtration rate were further categorized using predefined and clinically relevant cut-off values (>6 h, >15 points, and <60 mL/minute, respectively) [4,18,19].

### 2.3. Statistical Analysis

Normally distributed continuous variables were summarized using mean ± standard deviation, ordinal variables were summarized using median and interquartile range, and categorical variables were described using frequencies and percentages. In a univariate analysis, patients with and without AKI at 48 h were compared using either the Student’s T-test, Mann–Whitney U test, or Chi-square test, as appropriate. Independent predictors of AKI were then derived from a multiple logistic regression model, which included variables with a *p*-value < 0.20 on univariate analysis. The association between AKI and 3-month death or dependency was also assessed using logistic regression. For this purpose, crude Odds Ratios (ORs) were calculated using simple logistic regression and adjusted ORs were produced by fitting the same variables into the models. To minimize loss of precision and risk of bias due to missing observations, missing data on predictors were handled using multiple imputation with fully conditional specifications [20]. This method inputs missing data on a variable-by-variable basis using an imputational model for each incomplete variable and creates imputations in an iterative fashion, with the number of imputations being set at five. Values of *p* < 0.05 were considered statistically significant. Statistical analysis was performed using IBM SPSS statistics v 29.0.0.

### 2.4. Reporting

There was no protocol registration. We have reported this study according to the STROBE guidelines [21]. The corresponding author had full access to all data and takes responsibility for its integrity and the analysis.

## 3. Results

### 3.1. Study Patient Flow and Sample Characterization

**Figure 1:**
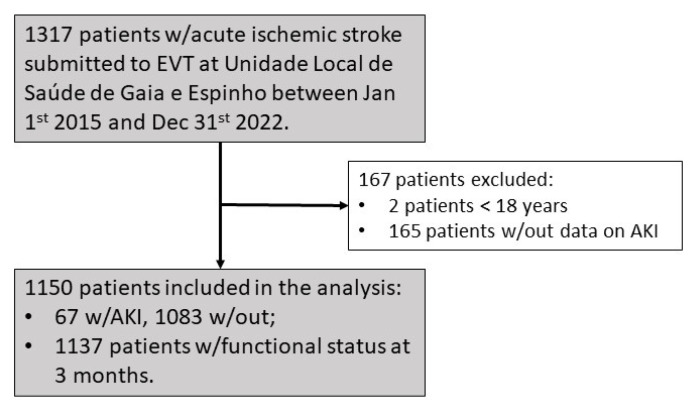
Flow diagram depicting inclusion of participants.

Figure 1 depicts the patient recruitment. Overall, 1317 patients fulfilled the inclusion criteria, but 165 had less than two creatinine quantifications in the first 48 h and two patients were under 18 years of age. The final sample size was, therefore, 1150. The study cohort’s mean age was 74 years and 56.2% were female (*n* = 647). The median NIHSS was 15 (IQR [9–19]), 90.7% of patients had anterior circulation stroke (*n* = 1045), and 45.1% were submitted to intravenous thrombolysis (*n* = 520). The mean onset-to-groin time was 392 min (IQR [193–485]) and 91.8% were successfully recanalized (*n* = 1043). Acute emergency stenting was required in 10.8% (*n* = 124). Hypertension, diabetes, dyslipidemia, and smoking were prevalent in 71.6%, 24.2%, 54.4%, and 12.8%, respectively (*n* = 822, 278, 625, and 147). Most strokes (53.9%) were cardioembolic (*n* = 621), with 15.5% originating in large vessel disease (*n* = 179) and 30.6% having other or undetermined causes (*n* = 352). The mean systolic and diastolic blood pressure values were 146.5 (±26.5) and 79.7 (±16.4) mmHg, the mean blood glucose was 139.6 (±54.7) mg/dL, and the mean GFR was 74.2 (±22.7) mL/min. Vital status and functional outcome information at three months was not available for 13 participants. The overall mortality rate at three months was 16.4% (*n* = 187), and 48.3% (*n* = 549) were dead or dependent at the same time frame. Data were missing regarding hypertension, diabetes, and dyslipidemia in four patients; smoking in six patients; NIHSS score in 15 patients; recanalization in 16 patients; onset-to-groin time in 20 patients; baseline glomerular filtration rate in 27 patients; systolic blood pressure in 213 patients; diastolic blood pressure in 215 patients; and blood glucose in 307 patients.

### 3.2. AKI Incidence and Predictors

Overall, 67 stroke patients submitted to EVT developed AKI at 48 h, with an estimated incidence rate of 5.8% (95% CI [4.5–7.3]). None of these patients required renal-replacement therapy and all of them were treated with supportive care, which consisted of correcting hypovolemia and excessive low blood pressure and avoiding exposure to nephrotoxic substances. Table 1 summarizes the descriptive statistics for patients with and without AKI, along with the univariate analysis of predictors. Patients with AKI were older (78.3 vs. 73.3 years), took longer time to EVT (482.5 vs. 386.5 min), and had more severe strokes (NIHSS 17 vs. 15), higher blood glucose (160.4 vs. 138.2 mg/dL), and lower baseline GFR (63.2 vs. 74.9 mL/min,). Patients with AKI also had a higher prevalence of dyslipidemia, although this difference did not reach statistical significance.

**Figure 2:**
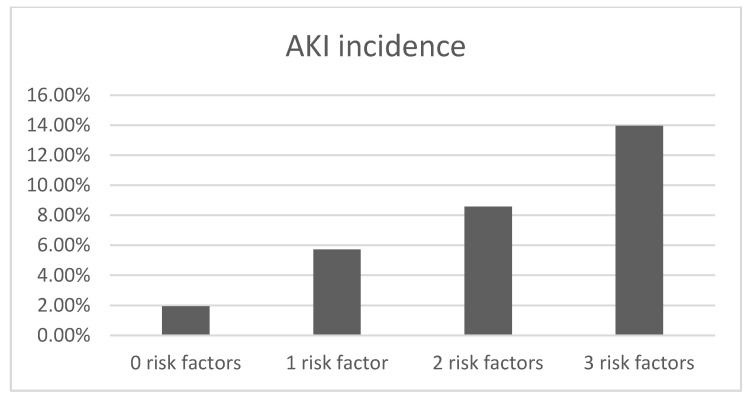
AKI incidence according to the number of risk factors present. Risk factors: baseline renal impairment (GFR < 60 mL/min), moderate to severe stroke (NIHSS > 15), and longer time to treatment (onset-to-groin time > 6 h).

The multivariate analysis of predictors of AKI after EVT for acute stroke is depicted in Table 2. After adjustment for potential confounders, AKI was independently associated with higher onset-to-groin time (Exp(β) = 1.001, *p* = 0.004), higher NIHSS (Exp(β) = 1.054, *p* = 0.012), and lower GFR (Exp(β) = 0.985, *p* < 0.001). When these variables are dichotomized using clinically meaningful cut-off values, the AKI incidence ranges from 1.9% in patients with no risk factors to 14.0% in patients with all three risk factors (Figure 2).

### 3.3. AKI and Stroke Outcome

**Figure 3:**
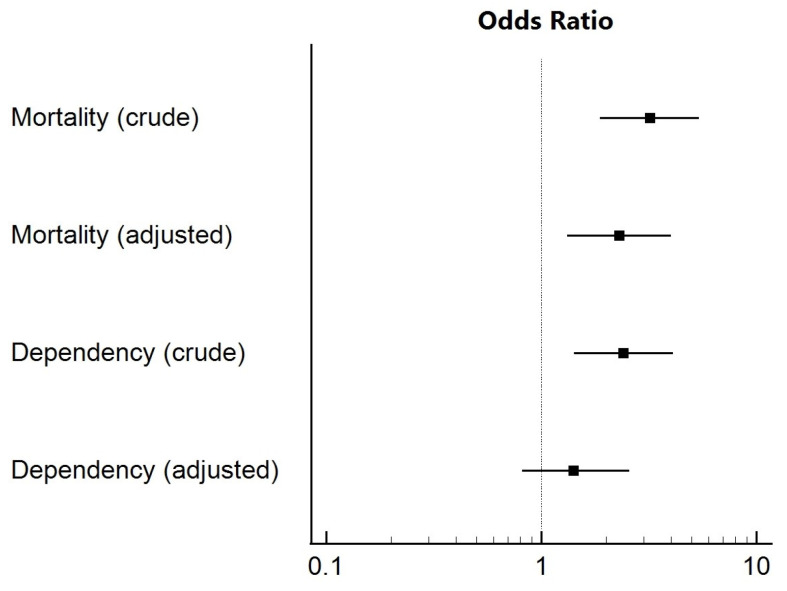
Association between AKI and mortality and dependency (modified Rankin score > 2) at 3 months after stroke (adjustment for age, onset-to-groin time, NIHSS score, dyslipidemia, blood glucose, and baseline glomerular filtration rate).

Figure 3 describes the association between AKI and stroke outcome at three months. In the univariate analysis, the development of AKI was associated with an increased risk of death (OR = 3.190, *p* < 0.001) and dependency (OR = 2.410, *p* = 0.001). After adjustment for potential confounders, AKI was associated with higher odds of death (OR = 2.302, *p* = 0.003) and a non-significant increase in dependency (OR = 1.415, *p* = 0.206).

## 4. Discussion

The incidence of AKI can vary significantly between studies, in part due to differences in the definition and timing of assessment [22]. In this study, we used the definition proposed by the Kidney Disease: Improving Global Outcomes (KDIGO) group assessed at 48 h [12], which demonstrated an incidence of 5.8%, meaning that, on average, 1 in every 17 stroke patients treated with EVT will develop this complication. These numbers are in accordance with a recent meta-analysis, which reported an incidence of between five and ten percent in this patient population [23], and lower than the incidence found in patients undergoing coronary angiography, which is estimated to be around 13% [24]. The difference in the latter is probably related to hemodynamic factors, which are more frequent in patients with coronary heart disease [25].

AKI is a clinical syndrome characterized by an abrupt decrease in renal function, reduced urinary output, retention of urea and other nitrogenous products, and imbalances in extracellular volume and electrolytes. This complication can be caused by a myriad of insults, ranging from renal hypoperfusion to nephrotoxicity and damage to the glomeruli, tubules, and interstitium [8]. By restricting the definition of AKI in our study to the first 48 h after EVT for stroke, we aimed to study a more homogeneous population and avoid potential confounding by clinical complications such as infections, sepsis, or arrhythmias.

**Figure 4:**
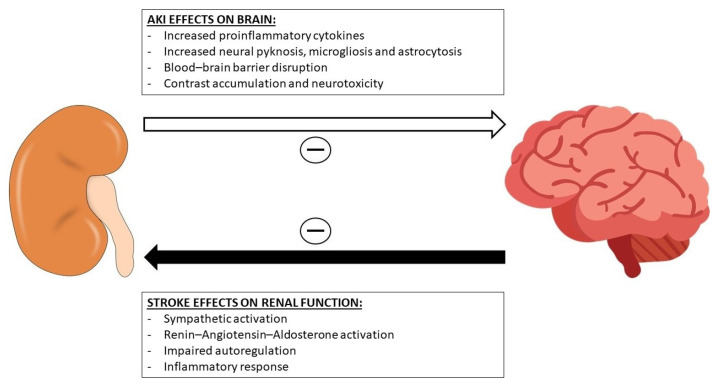
Association between AKI and stroke outcome and stroke severity with AKI.

The risk factor with the strongest association with AKI in our cohort was baseline renal impairment (*p* < 0.001), in accordance with several other studies in the literature [23]. Chronic kidney disease (CKD) and AKI were viewed in the past as distinct clinical entities, but are currently recognized as being closely related, whereby patients with CKD are less able to mount a complete adaptive response after acute renal injury, and instead repair in a maladaptive form with accelerated fibrosis and increased rate of renal decline. Hence, CKD is a risk factor for AKI and AKI contributes to CKD progression [26]. The other two risk factors for AKI identified in our study included stroke severity, as assessed by the NIHSS score (*p* = 0.012), and time to treatment (*p* = 0.004). Most studies have focused on the impact of AKI on clinical outcomes after stroke and reported a consistent association between AKI and worse prognosis [23]. Such an association was also present in our study, which showed that patients with AKI had a 2.3 times higher chance of death. Proposed mechanisms for this association include increased expression of pro-inflammatory cytokines in the brain, increased neural pyknosis, microgliosis, astrocytosis, and blood–brain barrier disruption [11]. Additionally, AKI can also reduce the renal excretion of contrast media, thereby contributing to contrast retention and direct contrast media neurotoxicity [27]. However, additional to the association between AKI and stroke prognosis, the present study also demonstrates that stroke severity, as assessed by the NIHSS score, and longer time to treatment are associated with development of AKI. Other authors have also found an association between stroke severity (higher NIHSS score, unsuccessful recanalization) and the development of AKI [28,29]. Several pathways have been suggested to mediate kidney dysfunction following acute stroke, involving the central autonomic pathway and sympathetic nervous system, inflammatory response, autoregulation, the neuroendocrine system, and extracellular vesicles [30]. Hence, a vicious cycle could therefore be established, whereby stroke severity begets AKI and AKI aggravates stroke prognosis and EVT outcome (Figure 4).

Regardless of the mechanisms involved, patients with baseline renal impairment, severe stroke, and longer time-to-EVT are particularly at risk for AKI, with incidence rates reaching values as high as 14% in patients with all three risk factors (Figure 2). It seems, therefore, sensible to monitor these patients well and take preventive actions by quickly expediting patients to treatment, avoiding nephrotoxic drugs, aggressively treating hypotension and volume depletion, and minimizing contrast exposure, preferably using iso-osmolar or low-osmolar contrast agents [12]. All of this should be carried out without delaying EVT, as this intervention has a tremendous impact on stroke outcome [31]. No drug has, so far, been proven to prevent contrast-induced nephropathy [7]. There is also no evidence to support that selecting patients for EVT using MRI instead of CT angiography and perfusion CT reduces AKI by minimizing ionic contrast exposure, as a systematic review with meta-analysis failed to show such benefit [31]. Of particular interest is the issue of hypotension and blood pressure control. Observational studies and meta-analyses of EVT-treated patients have demonstrated that increased systolic blood pressure in the first hours after mechanical thrombectomy is associated with increased chances of symptomatic hemorrhage and lower chances of functional recovery [32], but it remains unclear whether the relationship between high blood pressure and stroke outcome in these patients is linear or U-shaped [33]. Randomized controlled trials did not demonstrate a benefit of intensive blood pressure control, with more aggressive blood pressure reduction (below 120 mmHg) even leading to more dependency [34,35,36,37,38]. In a different patient population (intracerebral hemorrhage), the ATACH-2 trial demonstrated an increased incidence of renal events in patients submitted to aggressive blood pressure reduction [39]. Although speculative, as AKI was not defined as a safety event in the aforementioned thrombectomy trials, it is possible that AKI is, at least in part, responsible for the lack of benefit/harm from the intervention. Future trials should systematically assess this complication as part of the safety analysis.

There are limitations to this study that should be pointed out. First, we did not include low urinary output as part of the definition of AKI, as this variable was not available in our electronic health records. Although it is possible that we missed some cases of AKI according to the KDIGO definition (urinary output < 0.5 mL/kg/hour for at least 6 h) [12], studies have found that increases in serum creatinine are stronger predictors of adverse outcome than decreases in urinary output [40]. A potential explanation for this is that transient periods of oliguria might reflect insufficient volume repletion, whereas increases in serum creatinine might reflect established renal injury [41]. Based on this assumption, other studies have also omitted urinary output from the AKI definition [42]. Second, we did not include in the analysis information regarding the cause of AKI in the study participants. As stated earlier, AKI can be caused by a myriad of insults, including hemodynamic factors and the use of nephrotoxic substances. As a standard of care, all patients in our center undergo creatinine dosing prior to EVT and on the day after the procedure. By restricting AKI evaluation to the first 48 h after EVT, we minimized the effect of potential confounders such as medical complications or nephrotoxic drugs and ensured that AKI exposure was assessed the same way in all patients, regardless of baseline characteristics. Regarding hemodynamic factors, systolic and diastolic blood pressures were similar in presentation for both groups of patients (with and without AKI), but no data were available for blood pressure variation during the first hours after admission. As for nephrotoxic substances, patients at our stroke center are kept on IV fluids and nil per os in the first 24 h, with minimal drug exposure during this period consisting of IV fluids, insulin, and drugs for blood pressure and temperature control. The contrast agent used at our center during the study period was iopromide, but no data were available regarding the amount of contrast used in angio-CT scans and angiography. Although studies have demonstrated that contrast agents are a potential cause of acute kidney injury in the first days after treatment [43], other studies have failed to demonstrate a dose–effect relationship between the total dose of contrast given and the development of AKI [44]. Third, we did not include patients on chronic renal replacement therapy, as this treatment affects serum creatinine levels and, therefore, the assessment of AKI according to the criteria used in the study. We also did not include severely affected stroke patients requiring airway protection and ventilatory support, as these patients are cared for in a distinct intensive care unit at our hospital. With this in mind, by focusing on early AKI (<48 h), not identifying its cause, and recruiting stroke patients not requiring intensive care support on admission, we were not able to retrieve data on important outcomes such as ICU length of stay, time to extubation, sepsis-associated AKI, or dialysis requirement—outcomes that are relevant for severely affected patients. The study results cannot, therefore, be confidently applied this population.

Future studies should strive to ascertain the causes of AKI in EVT-treated stroke patients in order to correctly identify the potential mechanisms leading to AKI development and adverse stroke outcome. Such mechanisms are likely to differ according to the time course of the disease, with neurological insult and contrast nephropathy likely playing a more important role in the early stages (in the first 48 h) and other factors such as hypotension, sepsis, and drugs playing a role later on. For this purpose, complete data collection regarding all of these exposures is important [7]. Future studies should also explore novel biomarkers of acute kidney injury in this patient population [45]. Most studies so far have relied on serum creatinine levels to identify AKI in stroke patients [46], but serum creatinine is hampered by issues such as lack of baseline levels, interference by patient muscle mass and volume infusion and, most importantly, low sensitivity [47]. Newer biomarkers such as cystatin c, neutrophil gelatinase-associated lipocalin, interleukin-18, or kidney injury molecule-1 are not only more sensitive, but can potentially elucidate different pathways involved in AKI development due to their different insults, given their different nature and origin [45].

## 5. Conclusions

Acute kidney injury is a potential complication of endovascular therapy, affecting 1 in every 17 treated patients, on average, and is associated with worse outcomes. Patients with baseline renal impairment are particularly at risk, as are patients with more severe stroke (higher NIHSS and longer time-to-EVT). The latter suggests that not only can AKI aggravate neurological outcomes, but neurological injury can lead to AKI, creating a vicious cycle. More studies are required to clarify the mechanisms behind AKI development in this patient population, since the inciting factors can potentially differ between patients and according to the time course of the disease. Neurological injury and contrast administration probably play an important role in AKI development soon after stroke, whereas drug toxicity, hypotension, or sepsis can affect patients at a later period. Such separation can identify distinct subpopulations that require different strategies to prevent or mitigate the effects of AKI. In the meantime, preventive measures for patients at risk such as aggressively correcting hypovolemia, avoiding excessive reductions in blood pressure, minimizing exposure to contrast and other nephrotoxic substances, and quickly expediting patients to treatment seem appropriate.

## Figures and Tables

**Table 1:** Descriptive statistics for study participants with and without AKI and univariate analysis.

		AKI *n* = 67	No AKI *n* = 1083	*p*
Age	(years)	78.3 (±11.2)	73.3 (±12.8)	**0.002**
Sex	Male	27 (40.3%)	477 (44.0%)	0.360
	Female	40 (59.7%)	606 (56.0%)	
Thrombolysis		30 (44.8%)	488 (45.1%)	0.964
Circulation	Anterior	62 (92.5%)	981 (90.6%)	0.593
	Posterior	5 (7.5%)	102 (9.4%)	
Onset-to-groin time	(minutes)	482.5 (±369.0)	386.5 (±295.9)	**0.042**
Recanalization		63 (94.0%)	979 (91.8%)	0.508
NIHSS		17 [14–20]	15 [9–19]	**0.006**
Stenting		6 (9.0%)	118 (10.9%)	0.619
TOAST	Cardioembolic	37 (55.2%)	584 (53.9%)	0.926
	Large vessel	11 (16.4%)	168 (15.5%)	
	Other/Undetermined	19 (28.4%)	331 (30.6%)	
Hypertension		51 (76.1%)	771 (71.5%)	0.411
Diabetes		18 (26.9%)	260 (24.1%)	0.608
Dyslipidemia		44 (65.7%)	581 (53.8%)	0.059
Smoking		8 (11.9%)	139 (12.9%)	0.819
Blood glucose	(mg/dL)	160.4 (±75.0)	138.2 (±52.8)	**0.033**
SBP	(mmHg)	147.8 (±27.0)	146.4 (±26.5)	0.685
DBP	(mmHg)	79.8 (±17.4)	79.7 (±16.4)	0.948
Baseline GFR	mL/min/1.73 m^2^	63.2 (±21.7)	74.9 (±22.6)	**<0.001**
Good outcome		21 (31.8%)	567 (52.9%)	**<0.001**
Mortality		24 (36.4%)	163 (15.2%)	**<0.001**

**Table 2:** Results of multiple logistic regression analysis for the prediction of AKI at 48 h.

		Exp (β) [95%CI]	*p*
Age	(years)	1.014 [0.988–1.041]	0.297
Onset-to-groin time	(minutes)	1.001 [1.000–1.002]	**0.004**
NIHSS		1.054 [1.011–1.098]	**0.012**
Dyslipidemia		1.360 [0.797–2.318]	0.259
Blood glucose	(mg/dL)	1.003 [0.999–1.007]	0.094
Baseline GFR	mL/min/1.73 m^2^	0.985 [0.973–0.997]	**<0.001**

## Data Availability

The data that support the findings of this study are available from the corresponding author upon reasonable request.
